# Supplementary material for: Oleate Promotes Triple-Negative Breast Cancer Cell Migration by Enhancing Filopodia Formation through a PLD/Cdc42-Dependent Pathway
Source: Int J Mol Sci. 2024 Apr 2;25(7):3956. doi: 10.3390/ijms25073956 (PMC11012533; doi:10.3390/ijms25073956)
Supplement: Supplementary file 1 [file ijms-25-03956-s001.zip › ijms-2877419-supplementary.pdf]

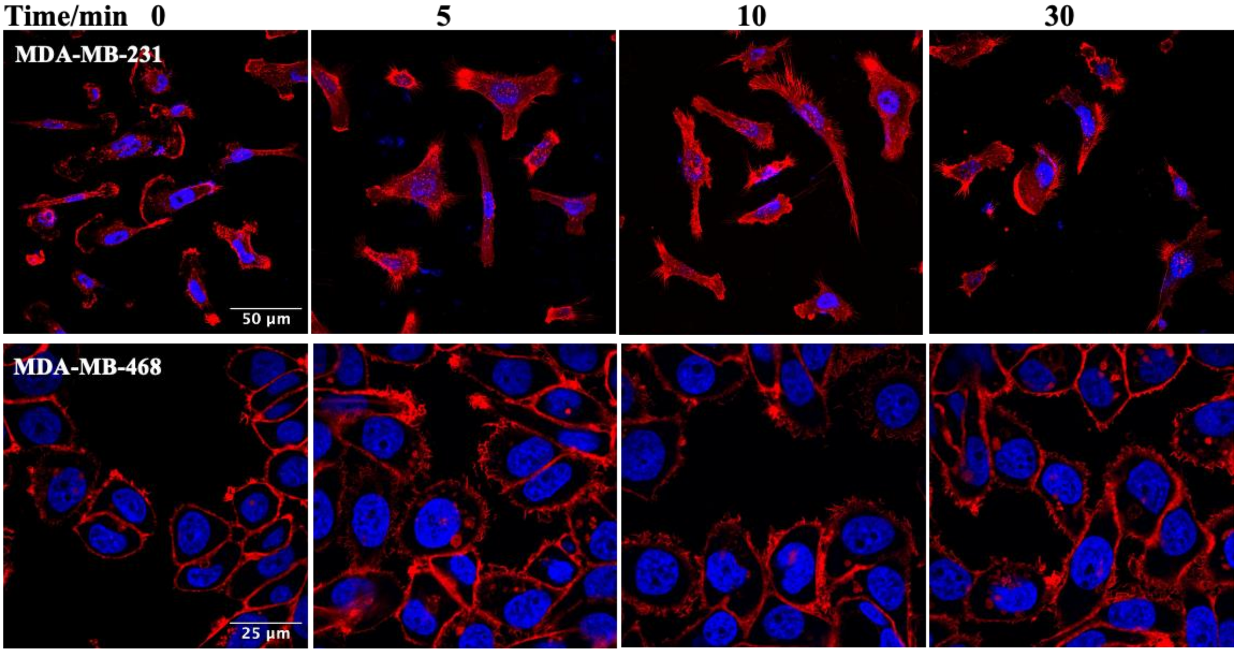

Figure S1. OA induced filopodia formation in TNBC cells. Representative confocal microscopy images of MDA-MB-231 and MDA-MB-468 cells treated with BSA (Ctrl) or 100 µM OA for indicated times and then stained with DAPI (blue) and Phalloidin-TRITC (red). The scale bars shown in the first images apply uniformly to all images within their set.

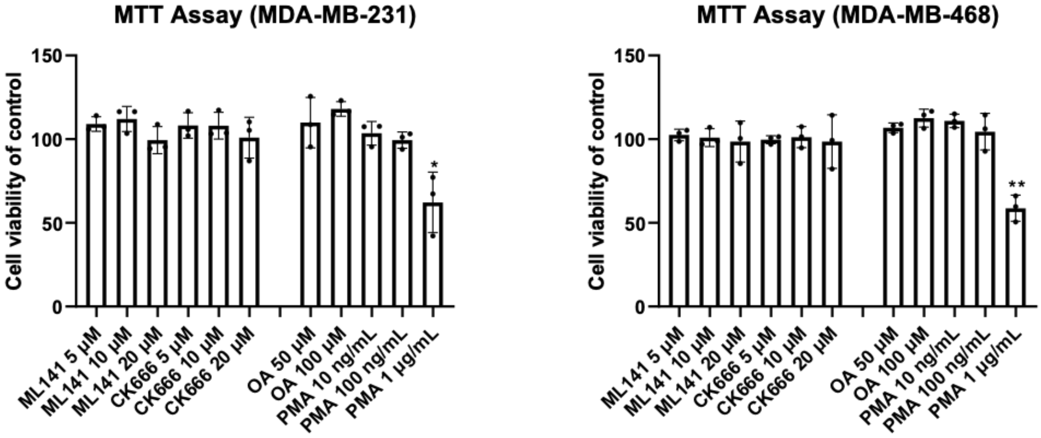

Figure S2. MTT assay in TNBC cells. Cell viability of MDA-MB-231 and MDA-MB-468 cells treated with Cdc42 (ML141) and Arp2/3 complex (CK666) inhibitors, or OA/PMA for 24 h at indicated concentrations. MTT assay values were normalized to a control group (treated with DMSO/BSA; 100%). The average (± standard deviation) percentage was calculated from 3 independent experiments. Statistical significance was determined comparing the treatment groups to their control groups (BSA or DMSO) via the student's t-test (*$P < 0.05$, **$P < 0.01$).

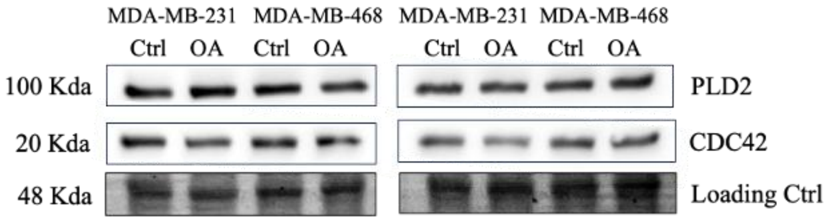

Figure S3. The impact of OA treatment on the expression of PLD2 and Cdc42 in TNBC cells. Protein expression of human PLD2 and Cdc42 in MDA-MB-231 and MDA-MB-468 cells treated with BSA (Ctrl) or 100 µM OA for 24 h. Loading control is provided as amido black.

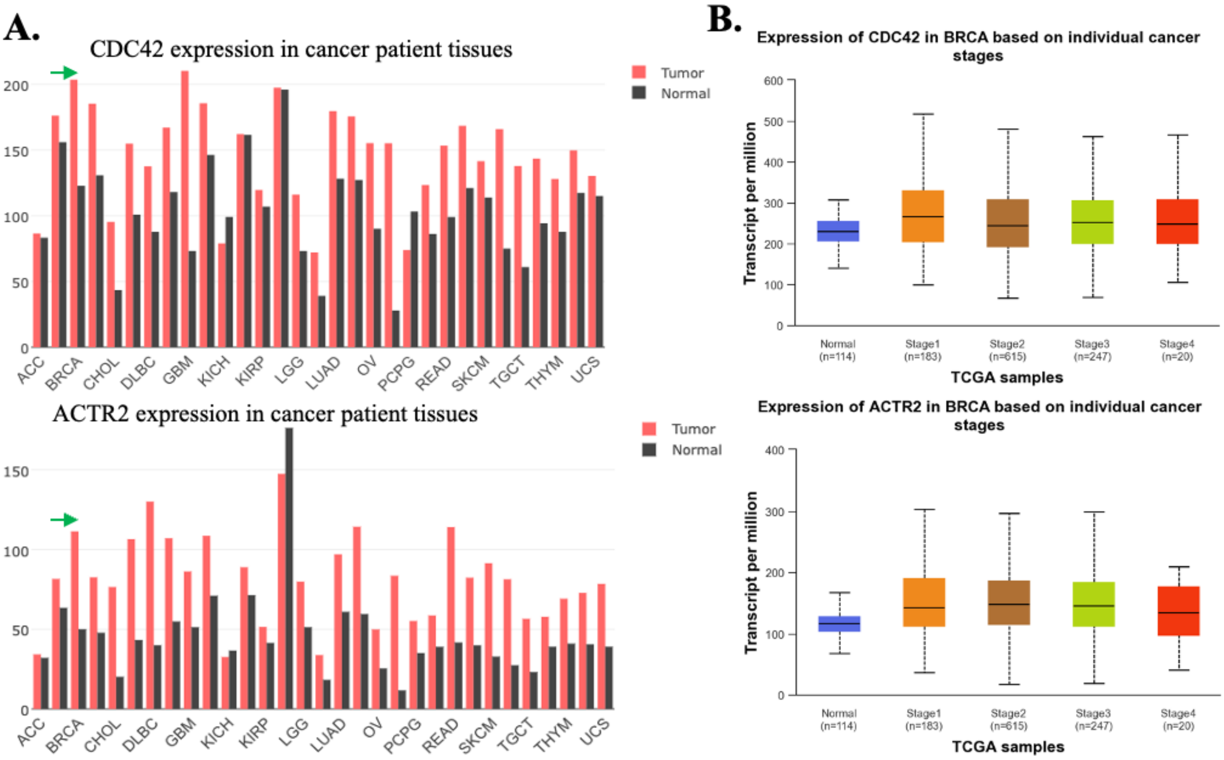

Figure S4. Gene expression profile of human CDC42 and ACTR2 in breast cancer patients. **(A)** Gene expression profile of human CDC42 and ACTR2 (Arp2 gene) across tumor (red) and paired normal (black) tissues. Bar plots were generated by GEPIA**.** The x-axis displays an array of cancer types, including ACC (adrenocortical carcinoma), BLCA (bladder urothelial carcinoma), BRCA (breast invasive carcinoma; highlighted with green arrows), CESC (cervical squamous cell carcinoma and endocervical adenocarcinoma), CHOL (cholangiocarcinoma), COAD (colon adenocarcinoma), DLBC (lymphoid neoplasm diffuse large b-cell lymphoma), ESCA (esophageal carcinoma), GBM (glioblastoma multiforme), HNSC (head and neck squamous cell carcinoma), KICH (kidney chromophobe), KIRC (kidney renal clear cell carcinoma), KIRP (kidney renal papillary cell carcinoma), LAML (acute myeloid leukemia), LGG (brain lower grade glioma), LIHC (liver hepatocellular carcinoma), LUAD (lung adenocarcinoma), LUSC (lung squamous cell carcinoma), MESO (mesothelioma), OV (ovarian serous cystadenocarcinoma), PAAD (pancreatic adenocarcinoma), PCPG (pheochromocytoma and paraganglioma), PRAD (prostate adenocarcinoma), READ (rectum adenocarcinoma), SARC (sarcoma), SKCM (skin cutaneous melanoma), STAD (stomach adenocarcinoma), TGCT (testicular germ cell tumors), THCA (thyroid carcinoma), THYM (thymoma), UCEC (uterine corpus endometrial carcinoma), UCS (uterine carcinosarcoma), UVM (uveal melanoma). **(B)** Gene expression of human CDC42 and ACTR2 across individual breast cancer stages. Bar plots were generated by UALAN.

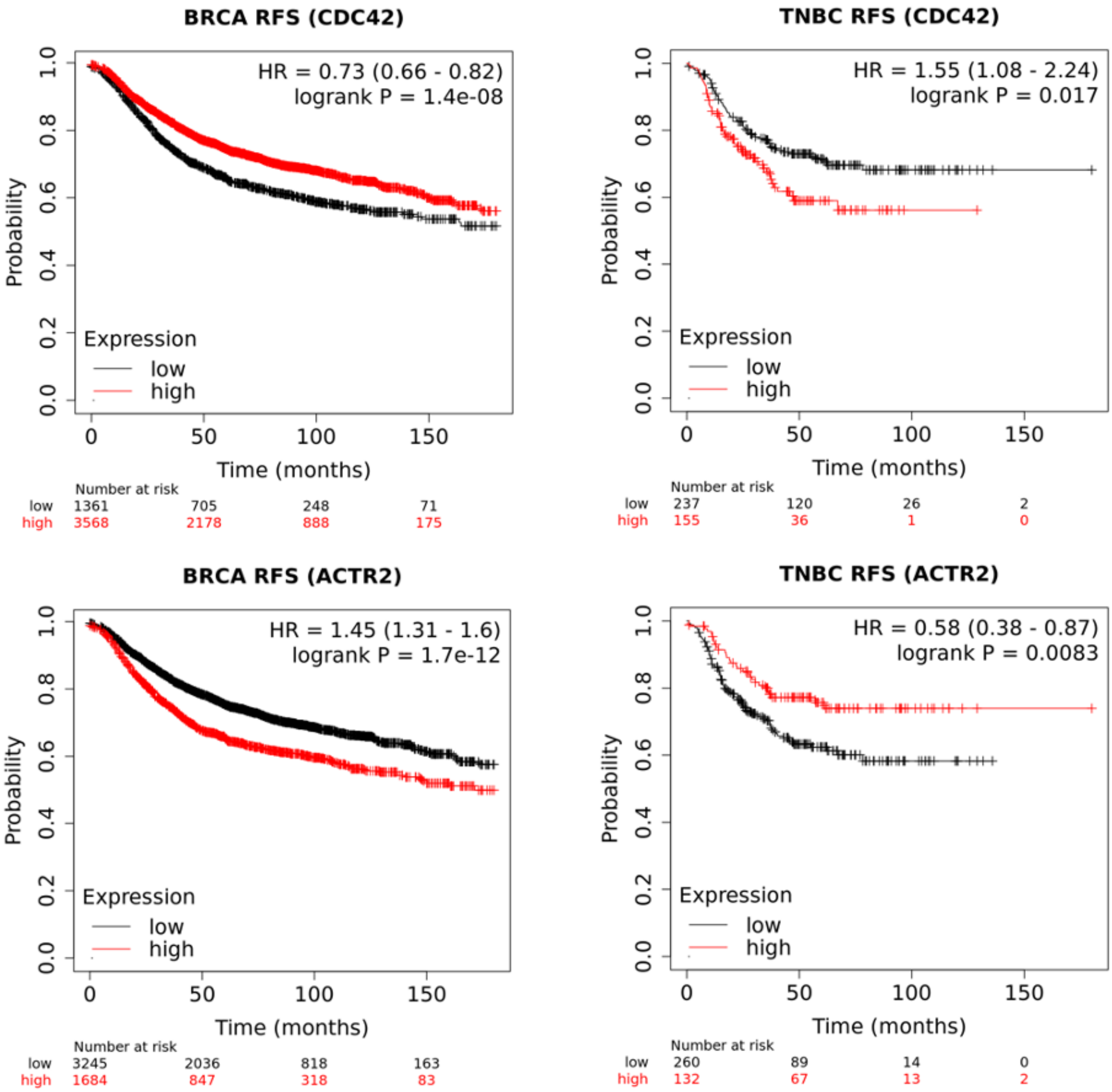

Figure S5. Kaplan–Meier survival plots of evaluation of relapse free survival (RFS) in breast cancer patients. All patients and TNBC subset analysis are over a span of up to 180 months, based on CDC42 and ACTR2 expression. "HR" stands for "Hazard Ratio", and "log-rank P" refers to the p-value obtained from a log-rank test.
